# AI-enhanced cancer radiotherapy quality assessment: utilizing daily linac performance, radiomics, dosimetrics, and planning complexity

**DOI:** 10.3389/fonc.2025.1503188

**Published:** 2025-03-13

**Authors:** Jia Deng, Yaolin Zhao, Dengdian Huang, Qingju Zhang, Ye Hong, Xiangyang Wu

**Affiliations:** ^1^ School of Nuclear Science and Technology, Xi’an Jiaotong University, Xi’an, Shanxi, China; ^2^ Radiation Oncology Department, Shaanxi Provincial Cancer Hospital, Xi’an, China; ^3^ Basic Technology Department, Science and Technology on Electromechanical Dynamic Control Laboratory, Xi’an, Shaanxi, China

**Keywords:** deep learning, radiotherapy, patient-specific quality assurance, prediction model, gamma passing rate

## Abstract

**Objective:**

This study aimed to develop and validate an Informer- Convolutional Neural Network (CNN) model to predict the gamma passing rate (GPR) for patient-specific quality assurance in volumetric modulated arc therapy (VMAT), enhancing treatment safety and efficacy by integrating multiple data sources.

**Methods:**

Analyzing 465 VMAT treatment plans covering head & neck, chest, and abdomen, the study extracted data from 31 complexity indicators, 123 radiomics features, and 123 dosimetrics indices, along with daily linac performance data including 141 key performance indicators. A hybrid Informer-CNN architecture was used to handle both temporal and non-temporal data for predicting GPR.

**Results:**

The Informer-CNN model demonstrated superior predictive performance over traditional models like Convolutional Neural Networks (CNN), Long Short-Term Memory(LSTM), and Informer. Specifically, in the validation set, the model achieved a mean absolute error (MAE) of 0.0273 and a root mean square error (RMSE) of 0.0360 using the 3%/3mm criterion. In the test set, the MAE was 0.0327 and the RMSE was 0.0468. The model also showed high classification performance with AUC scores of 0.97 and 0.95 in test and validation sets, respectively.

**Conclusion:**

The developed Informer-CNN model significantly enhances the prediction accuracy and classification of gamma passing rates in VMAT treatment plans. It facilitates early integration of daily accelerator performance data, improving the assessment and verification of treatment plans for better patient-specific quality assurance.

## 1 Introduction

The accuracy and safety of radiotherapy are critical factors influencing treatment outcomes for cancer patients (1, 2). Volumetric modulated arc therapy (VMAT), acknowledged as an advanced radiotherapy technique, provides significant benefits, especially in the treatment of complex tumors, owing to its precise and efficient dose distribution (3, 4). The aim of patient-specific quality assurance (PSQA) is to guarantee the safe delivery of treatment plans and enhance treatment outcomes by mitigating uncertainties and inaccuracies during plan execution (5, 6). While traditional PSQA methods, such as utilizing 2D or 3D diode arrays for physical dose measurement and comparing the outcomes with the planned dose distribution, have been extensively adopted, such approaches frequently demand considerable time and resources, particularly when managing complex IMRT/VMAT plans (7, 8). Furthermore, measurement-based methods often struggle to adapt quickly to the increasing complexity of modern radiotherapy plans, which may involve intricate tumor geometries and varying motion patterns.

These challenges can result in delays in plan validation, potentially limiting the ability to implement timely adjustments. Additionally, the dependency on physical measurements may not fully capture equipment-related variations, such as isocenter accuracy, MLC positioning errors, and absolute dose output, which are critical for ensuring overall treatment quality and consistency.

In recent years, computation-based PSQA methods have been increasingly adopted as they provide faster and more resource-efficient alternatives to traditional measurement-based approaches (9–13). For instance, Huang et al. (9) demonstrated the effectiveness of predicting dose distribution in virtual PSQA by employing the UNet++ architecture. Wall et al. (10) delved into the application of machine learning models, such as support vector machines (SVMs), for forecasting the QA outcomes of VMAT treatment plans. Expanding further, by prognosticating the gamma passing rate (GPR) of gated dosimetry using tree-based algorithms, Lam et al. (14) highlighted the utility of deep learning technology in enhancing the precision of radiotherapy QA. While recent research has achieved notable advancements, it remains constrained by certain limitations. Many studies primarily utilize data from radiotherapy planning and verification tools for training, which may not strongly correlate with the operational performance of radiotherapy linacs, including parameters such as isocenter accuracy, absolute dose, and MLC positioning error. In the present study, particular attention was paid to linac performance data. MPC daily check data were utilized (15), which records the daily status of radiotherapy equipment. Such data are crucial for monitoring and ensuring the stability and accuracy of treatment equipment. Further, traditional machine learning methods have largely been employed in existing research. However, the Informer model was introduced in the present study, which effectively captures long-term dependencies in time series data by leveraging attention mechanisms (16). This model is particularly suitable for analyzing time-dependent daily linac performance data in radiotherapy.

A spectrum of multi-dimensional features was harnessed in the present study, including linac performance data, radiomics, dosimetrics characteristics, and plan complexity, so as to develop an Informer-CNN model grounded in the Informer architecture. The primary objective is to facilitate the prompt acquisition of patient gamma pass rate information for VMAT treatment plans, leveraging daily linac performance data and treatment plans. This process enables rapid feedback and adjustments in the initial stages of radiotherapy, thereby enhancing the efficiency of treatment plan optimization. As such, this method not only ensures timelier quality assurance but also reduces the uncertainties in the VMAT plans clinically used.

## 2 Materials and methods

### 2.1 Data collection

#### 2.1.1 Radiation treatment plan

A total of 465 VMAT treatment plans (comprising 915 fields) from the years 2019 to 2023 were collected. All plans were generated using the Varian Eclipse planning system, employing a calculation grid of 2.5 mm and the AAA algorithm, with treatment energies of 6 MV and 6MV-FFF, administered via a Varian TrueBeam linac. The collected files included patient CT scans, treatment plans (RTPLAN), dose distributions (RTDOSE), and contour structures (RTSTRUCTURE). The information collected from the treatment plans is presented in **Table 1**.

**Table 1:** Plan characteristics.

Characteristic		Number of Plans	%	Number of Fields	%
Treatment site	Head & Neck	97	24.8	179	19.6
	Chest	145	31.2	288	31.5
	Abdomen	213	45.8	423	46.2
	Other	10	2.2	25	2.7
Energy	6MV	402	86.5	795	86.9
	6MV-FFF	63	13.5	120	13.1

#### 2.1.2 Dose verification data

Patient dose verification was conducted using the Portal Dosimetry software with settings of absolute gamma, normalization, and a threshold of 10%. Verifications were conducted with four different criteria: 3%3mm, 3%2mm, 2%2mm, and 1%1mm. The corresponding GPRs were collected, along with records of the verification times. The EPID panels employed were of the aSi1000 type, with dimensions measuring 40 × 30 cm². A backscatter absorption plate was positioned between the detection panel and the gantry. The detector matrix comprised 1024 × 768 pixels, providing a resolution of 0.39 mm (17). The GPRs distribution is presented in **Table 2**.

**Table 2:** The GPRs distribution.

Criteria	Number of Fields	Pass (Count)	Pass (Percentage)	Fail (Count)	Fail (Percentage)	Mean	Std Dev
3%/3mm	915	886	96.83	29	3.17	98.61	2.77
3%/2mm	915	849	92.79	66	7.21	97.53	4.25
2%/2mm	915	757	82.73	158	17.27	94.94	6.44
1%/1mm	915	29	3.17	886	96.83	72.43	13.28

#### 2.1.3 Daily machine data

Daily machine data measured between 2019 and 2023 were collected using the Varian’s Machine Performance Check (MPC) application. This tool, an EPID image-based application, was employed to assess the performance attributes of TrueBeam systems. The key performance metrics evaluated included mechanical isocenter deviation, multileaf collimator (MLC) leaf deviation, and beam uniformity deviation (18, 19).

### 2.2 Feature extraction

#### 2.2.1 Complexity of plan(C)

The complexity features of each beam in the plan were computed using the Pydicom package in Python 3.7, and these features were then extracted to be utilized as input for the model. A comprehensive set of 31 features was extracted from each treatment plan, encompassing both complexity-related attributes and other parameters such as the machine model, beam energy, MLC type, and jaw positions. The study included three Varian TrueBeam linacs with Millennium 120-leaf MLCs. The methodology employed for feature extraction follows the approach outlined by Dao Lam et al. (14) Detailed explanations of the features are provided in **Supplementary Table A1**.

#### 2.2.2 Radiomic (R) and dosimetric (D) features

Radiomic (R) and dosimetric (D) features were respectively extracted from the planning target volume (PTV) in computed tomography (CT) images. Utilizing the Pyradiomics open-source Python library (version 3.0), as outlined by van Griethuysen et al., 2017 (20), a total of 123 R features from pretreatment CT scans and 123 D features from RTDOSE files were extracted. Details of these extracted features can be found in **Supplementary Table A2**. To refine the feature extraction process, cavities within the PTV were eliminated from the original CT images by omitting CT values beneath -200 Hounsfield Units (HU), enhancing the precision in calculating R and D features. The threshold of -200 HU was chosen to exclude low-density regions, such as air cavities within the PTV, that are not representative of solid tissue. This ensures that R and D features are calculated based on clinically relevant areas, minimizing noise and enhancing the precision of feature extraction. The categories of extracted features included three-dimensional shape (exclusive to R, totaling 14 features), first-order statistics (comprising 18 features), and texture features such as the gray-level co-occurrence matrix (GLCM, 24 features), gray-level dependence matrix (GLDM, 14 features), gray-level run-length matrix (GLRLM, intended for 14 unique features), gray-level size zone matrix (GLSZM, 16 features), and neighboring gray tone difference matrix (NGTDM, 5 features). To enhance model interpretability, solely unfiltered raw images were utilized for extracting R and D features. This extraction was conducted within an image region delineated by a three-dimensional bounding box, strategically cropped with a voxel buffer of 10 voxels surrounding the PTV. To ensure consistency across datasets, both R and D features were standardized by discretizing voxel intensities using a bin width of 25 HU for radiomic features and 25 cGy for dosimetric features. These features contribute to GPR prediction by quantifying clinically relevant properties. For instance, GLCM features capture spatial texture patterns reflecting dose distribution consistency, while shape and first-order statistics provide information on PTV geometry and intensity, which are critical for understanding plan complexity and GPR outcomes. Three-dimensional shape features were derived solely from the structural geometry of the PTV in CT images, as they quantify anatomical characteristics such as volume, surface area, and compactness, which are clinically relevant for plan complexity and tumor characterization.

#### 2.2.3 Linac performance features(L)

Raw linac performance data were extracted from the MPC software and subsequently standardized to ensure uniformity across datasets. The MLC data underwent refinement to extract individual leaf information from the extensive dataset, facilitating a more granular performance analysis. A total of 141 daily performance features were extracted to provide an accurate depiction of the linear accelerator’s status. In addition, each data point was timestamped to facilitate the examination of performance variations over time. These processed and labeled datasets were subsequently organized for further analysis. **Supplementary Table A3** enumerates the performance status features of the linac.

### 2.3 Predictive model

#### 2.3.1 CNN

The Convolutional Neural Network (CNN) is a widely used architecture in deep learning that specializes in processing spatial data (21). It employs convolutional layers to extract hierarchical features, pooling layers for dimensionality reduction, and fully connected layers for final predictions. The CNN model comprises three convolutional layers with kernel sizes of 3×3, 5×5, and 3×3, respectively. Each layer employs ReLU activation, followed by average pooling (2×2 window) and dropout (rate=0.2) to mitigate overfitting. The final output is generated through a fully connected layer with ReLU activation, tailored to predict the four GPR metrics. The outputs include four GPR values (3%/3mm, 3%/2mm, 2%/2mm, 1%/1mm) and treatment success (GPR > 90%).

#### 2.3.2 LSTM

Long Short-Term Memory (LSTM) is an innovative design of recurrent neural network (RNN) tailored for sequential data processing (22). Unlike traditional RNN models, this architecture effectively tackles long-term dependency challenges by integrating three gating mechanisms alongside a dedicated memory unit. In contrast to standard RNNs, LSTMs are characterized by their utilization of a memory cell, which regulates the retention of information. The cell state forms the crux of the LSTM’s functionality. Within this memory cell, three distinct control gates—namely the input, forget, and output gates—are deployed to modulate and maintain the cell’s status. Each gate is structured around a neural network layer, which encompasses a sigmoid activation function and a point-wise multiplication operation. The LSTM model employs two stacked layers with 128 hidden units each, adopting the gating mechanisms and memory cell structure. This architecture, validated in sequential data tasks, ensures robust handling of temporal dependencies in radiotherapy QA parameters. The final LSTM layer connects to a dense layer with ReLU activation for GPR prediction.

#### 2.3.3 Informer

The Informer model is a supervised learning framework rooted in the attention mechanism, featuring both an encoder and a decoder (16). Built upon the Transformer architecture, it excels in capturing long-term dependencies inherent in time series data by incorporating additional steps such as position encoding, block attention, and adaptive length sequence sampling. The encoder’s role is to establish a robust understanding of the long-term dependencies within the original input sequences, while the decoder extends this understanding to predict future sequences. In this design, the encoder on the left-hand side handles longer input sequences and employs sparse self-attention, an enhanced version of the traditional self-attention mechanism. This self-attention refinement, effectively minimizes the network’s size and, when coupled with the layering of multiple levels, significantly bolsters the model’s strength. In contrast, the decoder on the right-hand side concentrates on long-term sequence inputs, disregarding irrelevant target elements, thereby enabling the assessment of attention-weighted features. Consequently, these elements are efficiently outputted. The encoder-decoder architecture employs two encoding layers with ProbSparse self-attention (256 hidden units, quad-head attention) followed by Feed-Forward Networks (FFN, 512-dimensional with ReLU activation). The FFN applies position-wise fully connected layers to transform attention outputs. The inclusion of all features ensures that both time-series and non-time-series data contribute to the prediction.

#### 2.3.4 Informer-CNN

**Figure 1:**
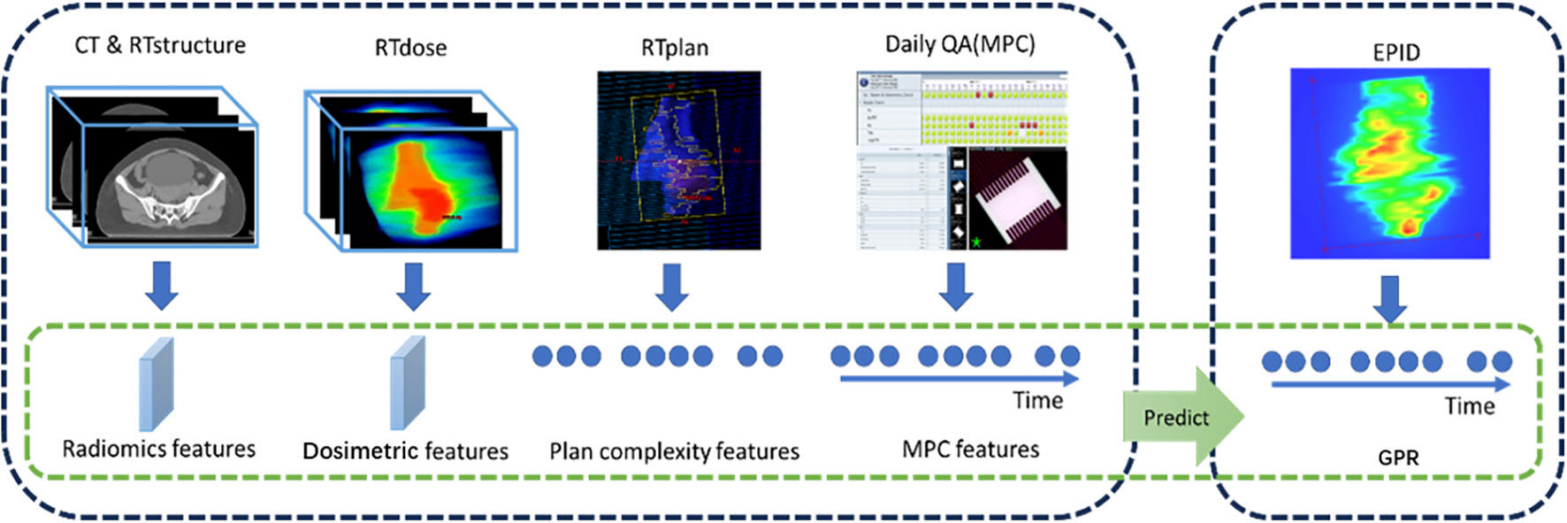
Model prediction workflow based on various feature extraction techniques.

**Figure 2:**
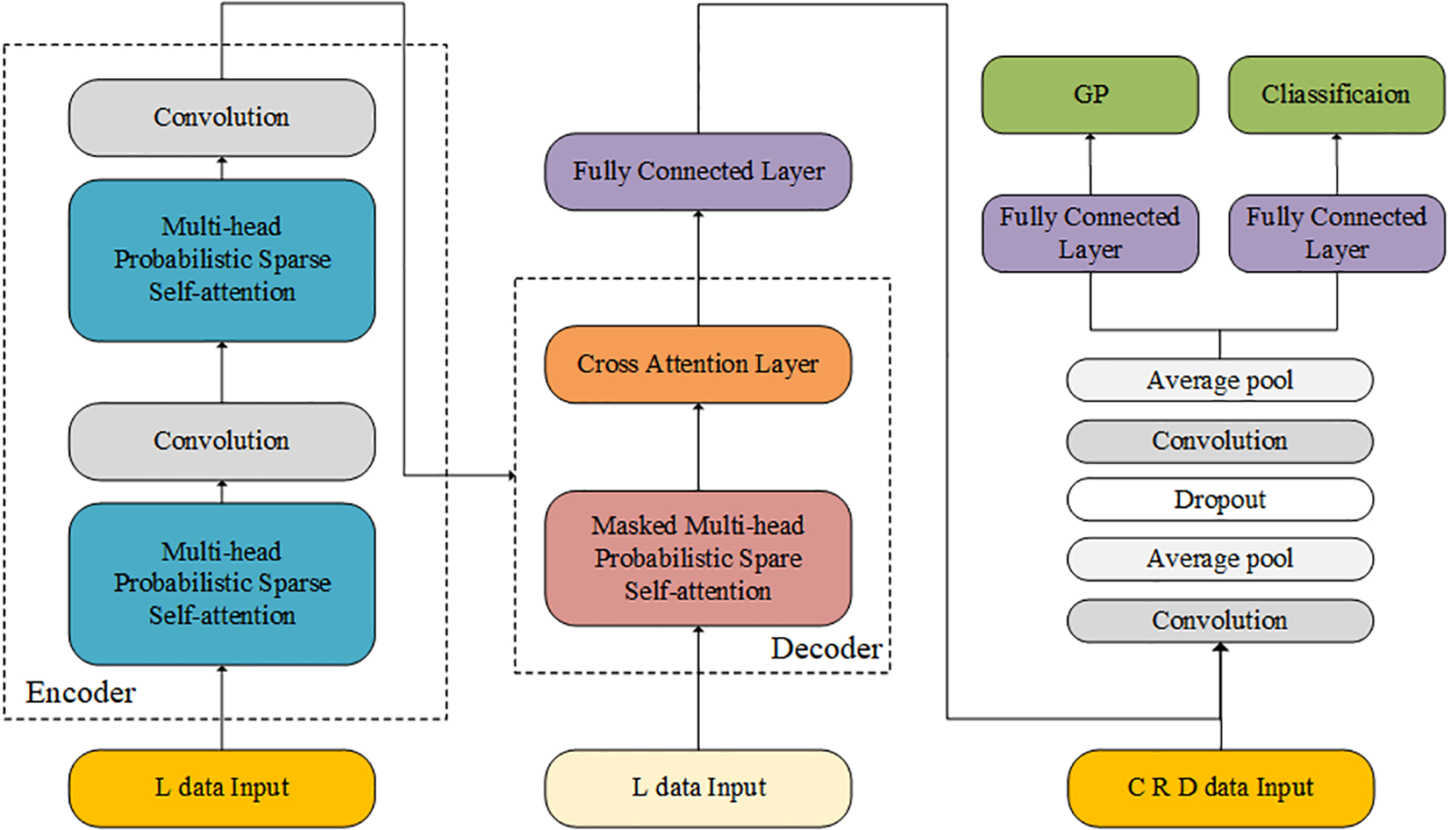
Architecture of the Informer-CNN used for prediction model.

In the present study, a deep learning framework based on the Informer model was developed for integrating time-series and non-time-series data to predict radiation therapy GPRs (see **Figures 1**, **2**). Data features were extracted from four sources: C (915×31), R (915×123), and D (915×123), L (915×141), and normalized using Z-score. The Informer architecture was adopted, with two encoding layers featuring the ProbSparse self-attention mechanism and 256 hidden units with quad-head self-attention. The Informer-CNN retains identical encoder-decoder parameters to the standalone Informer model, but appends CNN-based spatial feature processing to the decoder outputs. Following each self-attention layer, a 512-dimensional FFN was applied, incorporating layer normalization, residual connections, and downsampling, along with a dropout rate to mitigate overfitting. Subsequently, the encoded features underwent decoding, emphasizing previous outputs for precise future predictions. This process included self-attention and cross-attention layers, followed by FFN, layer normalization, and residual connections. Informer-processed features were then combined with other data, resulting in a dataset, which was then processed through convolutional layers, ReLU activation, average pooling, and dropout. The prediction module includes two parts: one predicts four GPR values (3%/3mm, 3%/2mm, 2%/2mm, 1%/1mm) using a fully connected layer with ReLU activation; the other predicts treatment success (GPR > 90%) using a fully connected layer with a Sigmoid function. The aim of such approach is to improve predictive accuracy in radiation therapy QA.

All models (CNN, LSTM, Informer, and Informer-CNN) utilize the full set of input features (C, R, D, L) and produce consistent outputs, including four GPR values (3%/3mm, 3%/2mm, 2%/2mm, 1%/1mm) and treatment success (GPR > 90%). This consistency ensures a direct comparison of performance across different architectures.

### 2.4 Model training and evaluation

In the present study, four models were generated and compared, including the CNN model, LSTM model, Informer model, and Informer-CNN model. The dataset of 465 VMAT treatment plans was split into training, validation, and testing sets using a random splitting method, with 70% allocated for training, 15% for validation, and 15% for testing. The network models were implemented using Python 3.7, on a 64-bit Windows operating system, equipped with 16.00 GB RAM and a 12th generation Intel(R) Core(TM) i7-12700KF processor at 3.60 GHz.

In this study, we trained a deep learning model using the Informer-CNN framework. The initial learning rate was set at 0.001, and we employed the Adam optimizer with settings of beta1 = 0.9, beta2 = 0.999, and epsilon = 1e-8. A validation set was used to fine-tune hyperparameters and prevent overfitting during model training. To prevent overfitting, we applied a learning rate decay of 0.95 every 20 epochs and set the batch size to 32. ReLU and Sigmoid activation functions were used in the convolutional and output layers, respectively. To mitigate potential data leakage, the validation and test sets were strictly isolated from the training process. Regularization techniques, including dropout (rate = 0.5) and L2 regularization (coefficient = 0.01), were applied alongside learning rate decay to ensure model generalization.

To evaluate the performance and accuracy of these models, various metrics were used for numerical prediction models: RMSE, MAE, and mean absolute percentage error (MAPE) (**Equations 1**–**3**). The classification prediction model used the ROC curve and the Area Under the Curve (AUC) metric (23). Among these, smaller values of RMSE, MAE, and MAPE indicate better predictive performance of the model. The calculation formulas are as follows, where $y_{i}$ represents the i-th actual value, ${\hat{y}}_{i}$ is the i-th predicted value, and N denotes the total number of observations (24):


(1) $$\text{RMSE}=\sqrt{\frac{1}{N}\sum_{i=1}^{N}{\left(y_{i}-{\hat{y}}_{i}\right)}^{2}}$$

(2) $$\text{MAE}=\frac{1}{N}\sum_{i=1}^{N}|y_{i}-{\hat{y}}_{i}|$$

(3) $$\text{MAPE}=\frac{100\%}{N}\sum_{i=1}^{N}|\frac{y_{i}-{\hat{y}}_{i}}{y_{i}}|$$

## 3 Results

### 3.1 Numerical prediction of GPR under different standards

**Figure 3:**
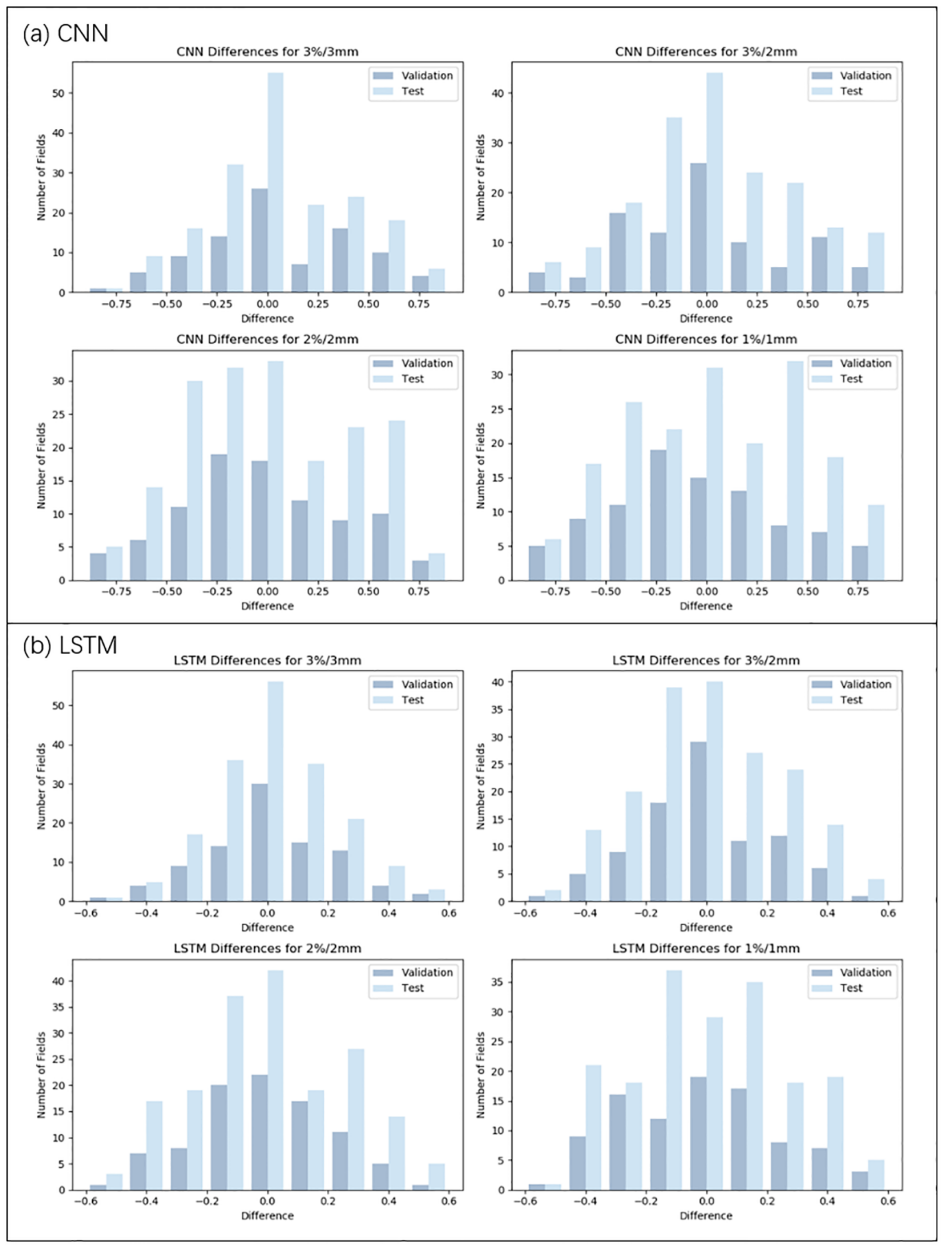
Distribution of prediction deviations for the CNN and LSTM model under four criteria (3%/3mm, 3%/2mm, 2%/2mm, and 1%/1mm). **(a)** Prediction deviation distribution of the CNN model. **(b)** Prediction deviation distribution of the LSTM model.

**Figure 4:**
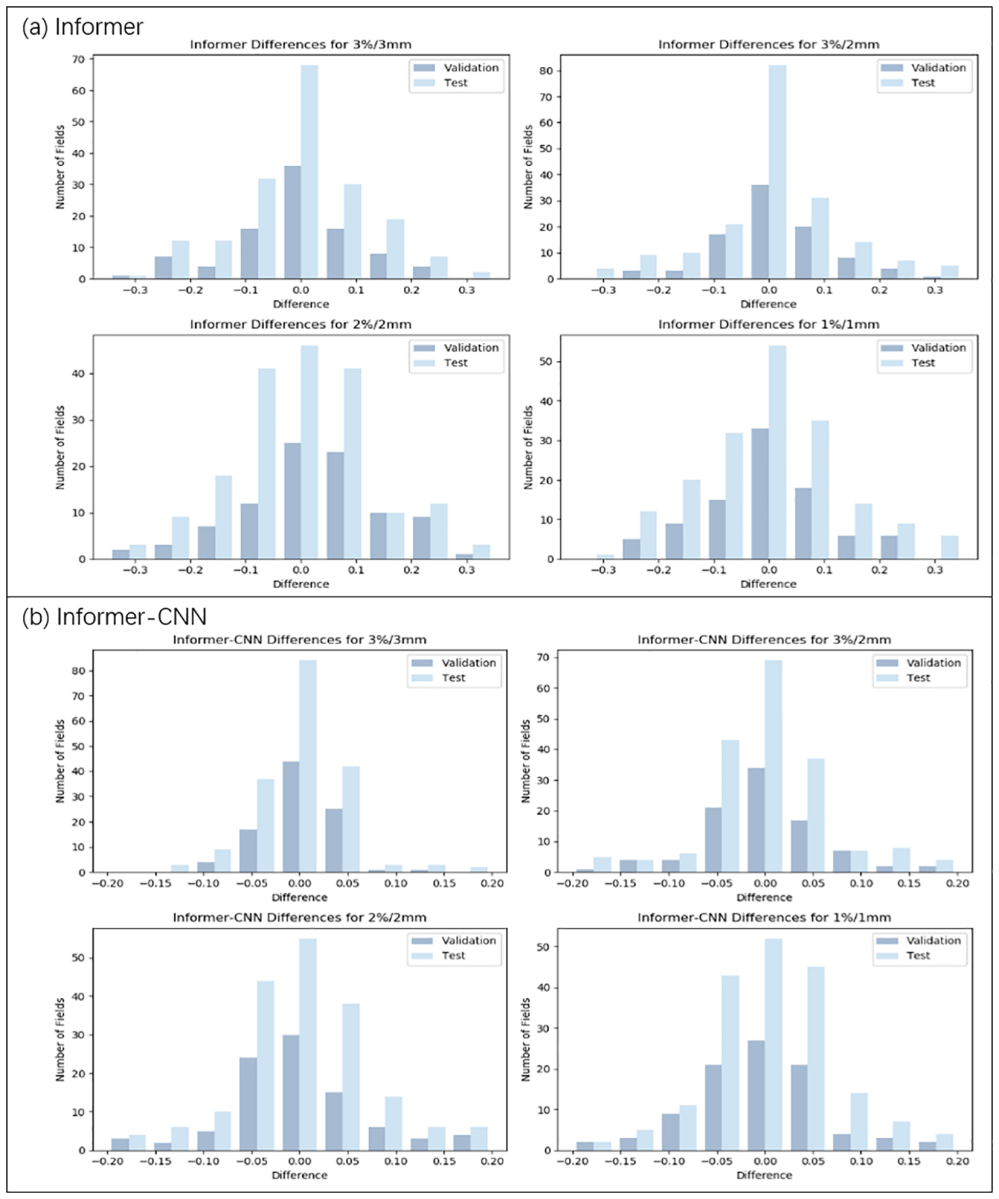
Distribution of prediction deviations for the Informer and Informer-CNN model under four criteria (3%/3mm, 3%/2mm, 2%/2mm, and 1%/1mm). **(a)** Prediction deviation distribution of the Informer model. **(b)** Prediction deviation distribution of the Informer-CNN model.

Numerical predictions for the GPR were performed using different models: CNN, LSTM, Informer, and Informer-CNN, for data training. The training and validation sets of these four models were assessed using RMSE, MAE, and MAPE, and the results are detailed in **Table 2**. Moreover, **Figures 3**, **4** depict the distributions of discrepancies between predicted values and actual values.

As illustrated in **Table 3**, **Figures 3**, **4**, under the 3%/3mm standard, the CNN validation metrics indicated a level of precision, with marginal improvement noted during the testing phase. The LSTM model exhibited a decrease in error rates (measured by RMSE, MAE, and MAPE) in both phases, suggesting enhanced accuracy. The Informer model demonstrated further improvement in these metrics, reflecting its effective management of complex data relationships. Notably, a reduction in error rates (in terms of RMSE, MAE, and MAPE) was observed in the Informer-CNN model compared to the other models, indicating its potential in predictive accuracy. This pattern was maintained under the 3%/2mm and 2%/2mm standards, with the Informer-CNN model generally outperforming the others, followed by Informer, LSTM, and CNN in that order. As the standards became more rigorous, an increase in error rates (in RMSE, MAE, and MAPE) was observed for all models, yet the relative order of their performance remained stable. Under the most stringent criterion, 1%/1mm, the hierarchy of performance remained consistent: the Informer-CNN model demonstrated the lowest error rates, succeeded by the Informer, LSTM, and CNN models, respectively.

**Table 3:** Comparative performance analysis of predictive models.

Standards	Metric	CNN		LSTM		Informer		Informer-CNN	
		Validation	Test	Validation	Test	Validation	Test	Validation	Test
3%/3mm	MAE	0.3008	0.2791	0.1717	0.1549	0.0837	0.0866	0.0273	0.0327
	MAPE	0.3050	0.2832	0.1740	0.1573	0.0849	0.0878	0.0278	0.0331
	RMSE	0.3782	0.3538	0.2190	0.2004	0.1141	0.1154	0.0360	0.0468
3%/2mm	MAE	0.3140	0.3154	0.1684	0.1901	0.0752	0.0824	0.0451	0.0447
	MAPE	0.3222	0.3250	0.1723	0.1952	0.0776	0.0849	0.0460	0.0459
	RMSE	0.3926	0.3912	0.2173	0.2352	0.1013	0.1157	0.0623	0.0633
2%/2mm	MAE	0.3336	0.3417	0.1788	0.2043	0.0986	0.0960	0.0518	0.0520
	MAP	0.3528	0.3613	0.1887	0.2153	0.1043	0.1005	0.0550	0.0549
	RMSE	0.4019	0.4056	0.2255	0.2504	0.1269	0.1230	0.0708	0.0695
1%/1mm	MAE	0.3452	0.3610	0.2049	0.2088	0.0854	0.0975	0.0506	0.0499
	MAPE	0.4911	0.5233	0.3026	0.2956	0.1193	0.1408	0.0723	0.0709
	RMSE	0.4145	0.4277	0.2460	0.2490	0.1121	0.1269	0.0675	0.0647

### 3.2 GPR classification prediction under different standards

**Figure 5:**
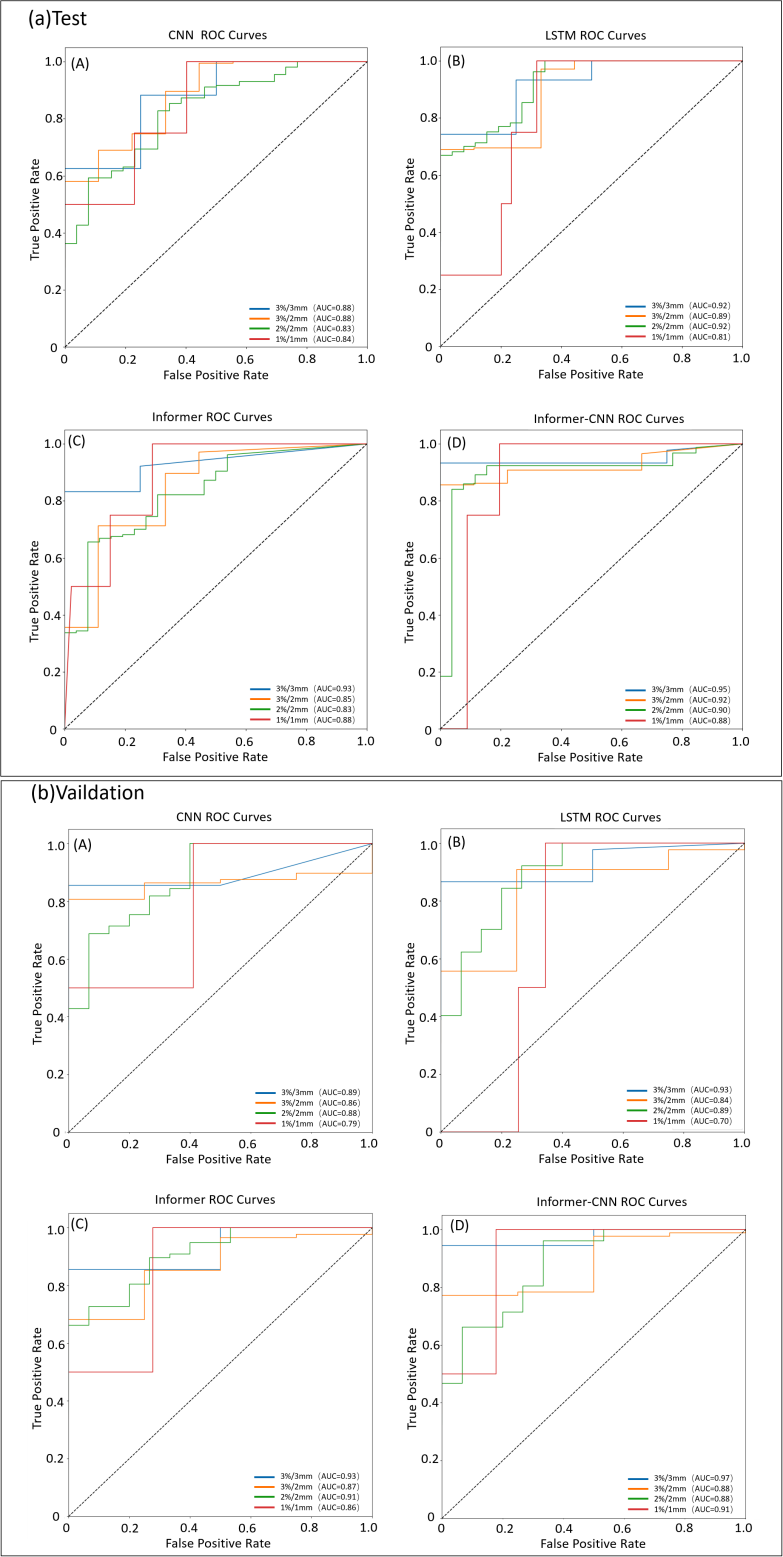
ROC curves for the test and validation set by different models under four criteria (3%/3mm, 3%/2mm, 2%/2mm, and 1%/1mm). **(a)** ROC curve for the test set. **(b)** ROC curve for the validation set.

GPR outcome classification predictions were conducted, with results above 90% representing a pass and those below 90% indicating a fail. The CNN, LSTM, Informer, and Informer-CNN models were utilized for data training. Evaluation was performed using the AUC and ROC curves, and the results are presented in **Table 4** and **Figure 5**.

**Table 4:** AUC values for GPR classification prediction by different models under four criteria.

Model	Group	3%/3mm	3%/2mm	2%/2mm	1%/1mm
CNN	Test	0.88	0.88	0.83	0.84
	Validation	0.89	0.86	0.88	0.79
LSTM	Test	0.92	0.89	0.92	0.81
	Validation	0.93	0.84	0.89	0.70
Informer	Test	0.93	0.85	0.83	0.88
	Validation	0.93	0.87	0.91	0.86
Informer-CNN	Test	0.95	0.92	0.90	0.88
	Validation	0.97	0.88	0.88	0.91

The CNN model exhibited comparable AUC values in both the testing and validation sets. Nevertheless, it demonstrated lower predictive accuracy, particularly under the stringent criterion of 1%/1mm. In contrast, the LSTM model generally surpassed the CNN model in the testing set, notably achieving higher AUC values, particularly at the 2%/2mm standard. Nevertheless, its AUC values decreased in the validation set, particularly under stricter standards, suggesting potential overfitting concerns. The Informer model exhibited higher AUC values in most standards for both testing and validation sets, notably improving performance under the 1%/1mm standard compared to other models. The Informer-CNN model consistently performed well across all standards, achieving the highest AUC values in both the testing and validation sets. Specifically, in the validation set under the 1%/1mm standard, an AUC of 0.91 was achieved, indicating high predictive accuracy and generalization capability.

## 4 Discussion

In the present study, an Informer CNN model based on the Informer architecture for long time series prediction was developed by integrating multi-modality features including linac performance, radiomics, dosimetrics, and plan complexity. PSQA gamma passing rate numerical and classification models for VMAT treatment plans under various criteria were developed. These models were then compared with CNN, LSTM, and Informer models, and their respective performances were evaluated.

Machine learning and deep learning methods have emerged as powerful quality assurance tools in radiotherapy, particularly for error detection and prevention, machine quality assurance, and patient-specific quality assurance. In the present study, particular attention was paid to the following multimodal features: linac performance status, radiomics, doseomics, and plan complexity. Initially, novel linac performance state features were developed to train the model. As per the recommendations outlined in the AAPM TG-218 report, if there are failures or issues detected during PSQA, it is imperative to review the linac’s daily and monthly QA procedures (25). This underscores the close relationship between the GPR measurements obtained during PSQA and the performance status of the linac on the given day. Notably, executing the same radiation plan at different times may yield varying measurement outcomes. The AAPM TG-218 report emphasized the crucial importance of regular QA of MLC in relation to PSQA. Hence, daily MPC data were employed, encompassing various indicators of linac performance status, including mechanical precision, dose accuracy, and MLC positioning accuracy, among others. Integrating such characteristics into the training of the prediction model can significantly enhance the precision and reliability of GPR prediction. The Informer model, distinguished for its applicability to long-term time series, was employed to accommodate the dynamic nature of MPC data. Emerging from the transformer architecture, the Informer model adeptly manages relational dynamics across different time points, thereby enhancing prediction accuracy. Its effectiveness has been extensively documented across various domains necessitating temporal predictions (26–28).

Further, the plan complexity feature is essential for predicting GPR. Lam et al. (11) achieved prediction accuracy with both AdaBoost and random forest algorithms, with 98% of predictions falling within 3% of the measured 2%/2 mm gamma pass rate and a mean absolute error of less than 1%. In addition, nine key plan complexity features (AAJA, MCS, MAD, EM, BI, MAXJ, BM, MSAS20, and MUCP) with a significant impact on prediction results were identified, underscoring the substantial relationship between plan complexity and GPR. Building on previous research, 31 plan complexity features were selected for analysis in the present study.

Moreover, radiomics and doseomics characteristics were extracted. Radiomics aims to quantify phenotypic features of medical imaging using automated algorithms, while dosimetrics focuses on quantifying phenotypic features of radiation dose distribution. Huang et al. (9) predicted GPR accurately by combining plan complexity and dosimetric features. The average MAE values for 3%/3mm, 3%/2mm, 2%/3mm, and 2%/2mm were 0.82, 0.88, 2.11, and 2.52, respectively. In addition, the AAPM-218 report (25) suggested the use of three-dimensional dose distribution to assess PSQA results. Radiomics and doseomics features offer insights into the spatial and volumetric dose distribution within the three-dimensional treatment volume (29), aiding in the identification of potential regions susceptible to underdose or overdose, which can affect the GPR. In addition, PTV delineation was utilized as a mask to obtain three-dimensional imaging information about tumors. Previous research has shown that radiomics and doseomics characteristics are related to the patient’s anatomical structure, radiotherapy dose distribution, and the GPR.

In constructing the model, to leverage the utilization of multimodal data during training, a CNN network was integrated with the Informer model. This fusion enhanced the model’s capacity to effectively process multimodal inputs (30). Two approaches for prediction were adopted: numerical and classification. The numerical prediction method can directly yield the GPR values, providing an intuitive understanding of the results. Conversely, classification prediction can determine whether a plan meets the quality assurance criteria, simplifying the evaluation into a pass or fail outcome. This dual-method approach aligns with the AAPM TG-218 report’s guidelines (25), which advocate for the use of tolerance and action limits in assessing GPR outcomes. Accordingly, the present study adheres to the recommended universal action limit of 90%, serving as the threshold for classifying results as either pass or fail.

In the numerical prediction of GPR, four models, CNN, LSTM, Informer and Informer CNN, were used. The performance of each model under different criteria was analyzed by evaluating the training and validation sets, including RMSE, MAE, MAPE, and error distribution. Under the evaluation criterion of 3%/3mm, the CNN model demonstrated basic predictive capabilities. However, the model’s performance on the test set showed limited improvement. In contrast, the LSTM exhibited a declining trend in error rates during both training and testing phases, suggesting its robust capability in processing and analyzing time-series data. The Informer model demonstrated further enhancements in predictive indicators, highlighting its exceptional capability in managing complex data relationships. Particularly noteworthy was the exceptional performance of the hybrid Informer-CNN model across multiple assessment metrics. At the 3%/3mm standard, it achieved an MAE of 0.0273 and an RMSE of 0.0360. Even under the most stringent standard of 1%/1mm, the model maintained commendable performance with an MAE of 0.0451 and an RMSE of 0.0623, indicating its substantial advantage in prediction accuracy. Such findings highlight the potential superiority of the Informer-CNN model in enhancing the precision of GPR data predictions. The outcomes of the present study surpassed those reported by Huang et al. (9), who employed the Unet++ model, which yielded a mean of 0.79 and a standard deviation of 1.28 at 3%3mm. Osman et al.’s (31) ANN model predicted an RMSE of 0.0097 mm for MLC position deviation. The present Informer CNN model was developed specifically to predict the GPR of VMAT treatment plans. While Osman et al. focused on MLC position accuracy, the prediction paradigm was broadened in the present study to include GPR results. The Informer CNN model effectively combines the advantages of Informer’s long-term dependency processing with CNN’s spatial feature extraction capability, offering a promising new approach to radiotherapy quality assurance.

In GPR classification prediction, the data were trained using CNN, LSTM, Informer, and Informer-CNN, and the results were evaluated using AUC and ROC curves. The Informer CNN model performed exceptionally well in GPR classification prediction, with AUC values of 0.97 and 0.95 in the test and validation sets, respectively. Cheng et al. (13) employed a combined model based on 1D complexity metrics and 3D plan dose to predict pretreatment PSQA results, with an AUC of 0.92 for QA classification. Glanville et al. (32) utilized a linear support vector classifier trained on treatment plan features and linac quality control metrics to predict VMAT patient-specific QA outcomes with an accuracy of 0.88. The model developed in the present study merges the long-term dependency processing capabilities of the Informer model with the spatial feature identification prowess of CNN. This synergy not only boosts the model’s capacity to handle multi-dimensional data features within VMAT treatment plans but also enhances its performance in terms of classification accuracy and generalization ability.

The prediction speed of the Informer-CNN model is noteworthy, operating at the second-level time scale per treatment plan. This efficiency highlights the model’s potential for providing timely feedback in clinical workflows. It is important to note that prediction time may vary depending on the computational resources available, and further optimization could enhance its performance. Unlike traditional PSQA methods that often require several minutes to hours for comprehensive evaluation, the Informer-CNN model provides predictions in a fraction of that time. This rapid feedback enables clinicians to make timely adjustments to treatment plans, thereby improving workflow efficiency and patient care outcomes. While random splitting may not preserve temporal dependencies, the proposed framework focuses on integrating heterogeneous features (time-series and non-time-series) under a controlled setup. Future studies will explore time-aware splitting to validate clinical applicability.

While the present study highlights the considerable promise of the Informer-CNN model in predicting the PSQA GPR for VMAT treatment plans, there are several limitations that must be addressed. The scope and variety of the case datasets employed in the present study were not extensive enough, potentially restricting a thorough assessment of the model’s generalization capabilities. Moreover, the focus of the study was on outcomes from specific linacs, suggesting that future efforts should encompass a broader array of linac models. Such expansion would contribute to the development of a more universally applicable prediction model, thereby enhancing its utility and precision in the realm of radiotherapy quality assurance.

## 5 Conclusion

The developed Informer-CNN model demonstrates superior prediction accuracy and classification of gamma passing rates in VMAT treatment plans compared to traditional models such as CNN, LSTM, and Informer alone. This model allows for early integration of daily accelerator performance data, ensuring more accurate assessment and verification of treatment plans for better patient-specific quality assurance.

## Data Availability

The raw data supporting the conclusions of this article will be made available by the authors, without undue reservation.
