# Supplementary material for: AI-enhanced cancer radiotherapy quality assessment: utilizing daily linac performance, radiomics, dosimetrics, and planning complexity
Source: Front Oncol. 2025 Mar 13;15:1503188. doi: 10.3389/fonc.2025.1503188 (PMC11966416; doi:10.3389/fonc.2025.1503188)
Supplement: Supplementary file 1 [file Table1.docx]

Supplementary Table A1: Complexity of Treatment plan Features

| Features | Explanation |
| --- | --- |
| BA | Beam aperture area weighted by MU |
| BI | Beam irregularity |
| BM | Fraction of BA normalized by UAA |
| UAA | Union area of aperture (UAA) |
| MFAS_2,5,10,20_ | Mean of fraction of aperture smaller (MFAS) than 2, 5, 10, 20 mm |
| MaxFAS_2,5,10,20_ | Max of fraction of aperture smaller (MaxFAS) than 2, 5, 10, 20 mm |
| MAA | Mean aperture area |
| MAD | Maximum distance of the mid-point between any open leaf-pair in a beam |
| MUCP | Mean of MUs per control point in a beam |
| MLO_1,2,3,4,5_ | Moment order of 1, 2, 3, 4, 5 of leaf openings |
| minAP_h | Minimum aperture perimeter in horizontal direction |
| maxAP_h | Maximum aperture perimeter in horizontal direction |
| minAP_v | Minimum aperture perimeter in vertical direction |
| maxAP_v | Maximum aperture perimeter in vertical direction |
| maxRegs | Maximum number of regions in the beam |
| AAJA | Ratio of the average area of an aperture over the area defined by jaws |
| MAXJ | Maximum of x-y jaw positions |
| MCS | Modulation complexity score |
| EM | Edge metric: ratio of MLC side-length to aperture area |
| Energy | Beam energy |
| MU |  |

Supplementary Table A2: Radiomic (R) and dosimetric (D) features

| Feature Class | Feature Name | No. of Features |
| --- | --- | --- |
| Shape (S) | Elongation, Flatness, LeastAxisLength, MajorAxisLength, Maximum2DDiameterColumn, Maximum2DDiameterRow, Maximum2DDiameterSlice, Maximum3DDiameter, MeshVolume, MinorAxisLength, Sphericity, SurfaceVolumeRatio, VoxelVolume | 14 |
| First-order (L1) | InterquartileRange, Skewness, Uniformity, Median, Energy, RobustMeanAbsoluteDeviation, MeanAbsoluteDeviation, TotalEnergy, Maximum, RootMeanSquared, 90Percentile, Minimum, Entropy, Range, Variance, 10Percentile, Kurtosis, Mean | 18 |
| Second-order (L2) | JointAverage, SumAverage, JointEntropy, ClusterShade, MaximumProbability, Idmn, JointEnergy, Contrast, DifferenceEntropy, InverseVariance, DifferenceVariance, Idn, Idm, Correlation, Autocorrelation, SumEntropy, MCC, SumSquares, ClusterProminence, Imc2, Imc1, DifferenceAverage, Id, ClusterTendency | 24 |
| Gray level dependence matrix (GLDM) | GrayLevelVariance, HighGrayLevelEmphasis, DependenceEntropy, DependenceNonUniformity, GrayLevelNonUniformity, SmallDependenceEmphasis, SmallDependenceHighGrayLevelEmphasis, DependenceNonUniformityNormalized, LargeDependenceEmphasis, LargeDependenceLowGrayLevelEmphasis, DependenceVariance, LargeDependenceHighGrayLevelEmphasis, SmallDependenceLowGrayLevelEmphasis, LowGrayLevelEmphasis | 14 |
| Higher-order (L3) | GrayLevelVariance, SmallAreaHighGrayLevelEmphasis, GrayLevelNonUniformityNormalized, SizeZoneNonUniformityNormalized, SizeZoneNonUniformity, GrayLevelNonUniformity, LargeAreaEmphasis, ZoneVariance, ZonePercentage, LargeAreaLowGrayLevelEmphasis, LargeAreaHighGrayLevelEmphasis, HighGrayLevelZoneEmphasis, SmallAreaEmphasis, LowGrayLevelZoneEmphasis, ZoneEntropy, SmallAreaLowGrayLevelEmphasis | 16 |
| Gray level run length matrix (GLRLM) | ShortRunLowGrayLevelEmphasis, GrayLevelVariance, LowGrayLevelRunEmphasis, GrayLevelNonUniformityNormalized, RunVariance, GrayLevelNonUniformity, LongRunEmphasis, ShortRunHighGrayLevelEmphasis, RunLengthNonUniformity, ShortRunEmphasis, LongRunHighGrayLevelEmphasis, RunPercentage, LongRunLowGrayLevelEmphasis, RunEntropy, HighGrayLevelRunEmphasis, RunLengthNonUniformityNormalized | 16 |
| Gray level size zone matrix (GLSZM) | GrayLevelVariance, SmallAreaHighGrayLevelEmphasis, GrayLevelNonUniformityNormalized, SizeZoneNonUniformityNormalized, SizeZoneNonUniformity, GrayLevelNonUniformity, LargeAreaEmphasis, ZoneVariance, ZonePercentage, LargeAreaLowGrayLevelEmphasis, LargeAreaHighGrayLevelEmphasis, HighGrayLevelZoneEmphasis, SmallAreaEmphasis, LowGrayLevelZoneEmphasis, ZoneEntropy, SmallAreaLowGrayLevelEmphasis | 16 |
| Neighboring gray tone difference matrix (NGTDM) | Coarseness, Complexity, Strength, Contrast, Busyness | 5 |

Supplementary Table A3: Lina performance status features

| Feature Class | Feature Name |
| --- | --- |
| Isocenter | Size ,MV imager projection offset, kV imager projection offset |
| Collimation | Maximal offset leaves A, Maximal offset leaves B, Mean offset leaves A, Mean offset leaves B, Individual MLC leaf A, Individual MLC leaf B, Jaw X1, Jaw X2, Jaw Y1, Jaw Y2, Rotation offset |
| Gantry | Absolute, Relative |
| Couch | Lateral, Longitudinal, Pitch, Roll, Rotation, Vertical, Rotation-induced couch shift |
| Kilovolt imager | In-plane rotation, Source axial, Tangential |
| Megavolt imager | In-plane rotation, Source Axial, Tangential |
| Beam | Center shift, Beam output change, Uniformity change |
